# Single-Cell RNA Sequencing Reveals the Role of Phosphorylation-Related Genes in Hepatocellular Carcinoma Stem Cells

**DOI:** 10.3389/fcell.2021.734287

**Published:** 2022-01-04

**Authors:** Fuwen Yao, Yongqiang Zhan, Changzheng Li, Ying Lu, Jiao Chen, Jing Deng, Zijing Wu, Qi Li, Yi’an Song, Binhua Chen, Jinjun Chen, Kuifeng Tian, Zuhui Pu, Yong Ni, Lisha Mou

**Affiliations:** ^1^ Department of Hepatopancreatobiliary Surgery, Shenzhen Institute of Translational Medicine, Health Science Center, The First Affiliated Hospital of Shenzhen University, Shenzhen Second People’s Hospital, Shenzhen, China; ^2^ Shenzhen Xenotransplantation Medical Engineering Research and Development Center, Shenzhen Institute of Translational Medicine, Health Science Center, The First Affiliated Hospital of Shenzhen University, Shenzhen Second People’s Hospital, Shenzhen, China; ^3^ Key Laboratory of Stem Cells and Tissue Engineering, Zhongshan School of Medicine, Sun Yat-sen University, Ministry of Education, Guangzhou, China; ^4^ Imaging Department, Shenzhen Institute of Translational Medicine, Health Science Center, Shenzhen Second People’s Hospital, The First Affiliated Hospital of Shenzhen University, Shenzhen, China

**Keywords:** AURKA, EZH2, tyrosine kinase inhibitors, TKI, protein kinases, cell cycle, single-cell RNA sequencing, hepatocellular carcinoma

## Abstract

Abnormal activation of protein kinases and phosphatases is implicated in various tumorigenesis, including hepatocellular carcinoma (HCC). Advanced HCC patients are treated with systemic therapy, including tyrosine kinase inhibitors, which extend overall survival. Investigation of the underlying mechanism of protein kinase signaling will help to improve the efficacy of HCC therapy. Combining single-cell RNA sequencing data and TCGA RNA-seq data, we profiled the protein kinases, phosphatases, and other phosphorylation-related genes (PRGs) of HCC patients in this study. We found nine protein kinases and PRGs with high expression levels that were mainly detected in HCC cancer stem cells, including *POLR2G*, *PPP2R1A*, *POLR2L*, *PRC1*, *ITBG1BP1*, *MARCKSL1*, *EZH2*, *DTYMK,* and *AURKA.* Survival analysis with the TCGA dataset showed that these genes were associated with poor prognosis of HCC patients. Further correlation analysis showed that these genes were involved in cell cycle-related pathways that may contribute to the development of HCC. Among them, *AURKA* and *EZH2* were identified as two hub genes by Ingenuity Pathway Analysis. Treatment with an AURKA inhibitor (alisertib) and an EZH2 inhibitor (gambogenic) inhibited HCC cell proliferation, migration, and invasion. We also found that both *AURKA* and *EZH2* were highly expressed in *TP53*-mutant HCC samples. Our comprehensive analysis of PRGs contributes to illustrating the mechanisms underlying HCC progression and identifying potential therapeutic targets for future clinical trials.

## Introduction

The most frequent type of primary liver cancer is hepatocellular carcinoma (HCC) (Budny et al., 2017; Khemlina et al., 2017). It contains approximately 85% of cirrhosis cases and is currently the sixth-leading cause of cancer worldwide (Asafo-Agyei and Samant, 2021) According to GLOBOCAN 2020 statistics, the estimated liver cancer cases summed up to 905,677 and deaths to 830,180 in 2020 worldwide (Sung et al., 2021). HCC commonly occurs in patients with chronic liver diseases such as hepatitis B and C infection (El-Serag, 2012). Treatments such as excision or transplantation may be successful in the early stages of HCC, but only a few therapeutic options are available when advanced HCC develops (Lencioni et al., 2014).

The dysregulation of phosphorylation-dependent signaling pathways is a hallmark of oncogenesis (Bhatia et al., 2010; Berndt et al., 2013; Liu et al., 2018). Early this century, the first tyrosine kinase inhibitor (TKI) was approved as a potential precision therapy for HCC (da Fonseca et al., 2020). It was more efficient and safer than traditional chemotherapies because it was more specific to target and had a lower impact on normal cells (Forner et al., 2018). TKIs are used for patients with advanced unresectable HCC and have proven clinically efficacious (Wei et al., 2019; Lee et al., 2020). However, TKI resistance has become a great concern in recent years (Dal Bo et al., 2020; Huang et al., 2020). An example would be the treatment failure of sorafenib due to the high-level inter-tumoral and intra-tumoral heterogeneity (Cabral et al., 2020). Interrogating the underlying molecular basis of abnormal expression of protein kinases, phosphatases, and other phosphorylation-related genes (PRGs) will help to improve the efficacy of HCC therapy.

Cancer stem cells (CSCs) were identified and considered as one of the most important reasons for heterogeneity in HCC, generating diverse cell populations and leading to the selection of the tumor microenvironment (Yin et al., 2010; Eun et al., 2017; Fujii and Sato, 2017; Zhu et al., 2018). The resistance caused by CSCs to TKIs is an obstacle to the total eradication of carcinoma (Graham et al., 2002). Previous studies reported that abnormal phosphorylation was linked to the capacity of CSCs to proliferate and differentiate (Cianflone et al., 2020). However, the expression characteristics of all PRGs in CSCs of HCC have yet to be revealed. Recent high-throughput approaches of single-cell RNA sequencing (scRNA-seq) allow for a better understanding of tumor heterogeneity and transcriptional plasticity in HCC, which may provide additional insight to improve the efficacy of HCC therapy (Kim et al., 2018; Zheng et al., 2018; Aizarani et al., 2019; Zhang et al., 2019a; Kang et al., 2019; Ma et al., 2019). ScRNA-seq provides a viable strategy for the elucidation of abnormal phosphorylation-dependent signaling pathways in multiple cell types of tumors, especially CSCs, which could help to identify new effective drugs in the therapy of HCC.

In this study, we identified 18 PRGs involved in HCC initiation and progression based on The Cancer Genome Atlas (TCGA) RNA-Seq data. In addition, scRNA-seq data of HCC patients provides further evidence that nine of these PRGs were expressed in CSCs and correlated with tumor progression. We performed cell cycle inference analysis of PRGs in CSCs of HCC and found that they played crucial roles in cell cycle transition. Among these genes, Aurora kinase A (*AURKA*) and Enhancer of Zeste 2 (*EZH2*, Polycomb Repressive Complex 2 Subunit) were correlated with multiple cell cycle genes and may take part in cell cycle transition. HCC cell lines treated with an *AURKA* inhibitor (alisertib) or *EZH2* inhibitor (gambogenic acid) exhibited impeded cell proliferation and decreased clone formation, migration, and invasion capacities. Taken together, our results provide a framework for deeper investigation into PRGs in HCC patients. Alisertib and gambogenic acid, which act as inhibitors of *AURKA* and *EZH2*, hold promise as novel therapeutic drugs for HCC.

## Materials and Methods

### Data Collection and Preprocessing

The gene expression data of 369 HCC patients and 50 adjacent cancer samples and clinical data of matched patients were obtained from The Cancer Genome Atlas (TCGA) data portal (https://TCGAData.nci.nih.gov/TCGA/). Fragments per kilobase million (FPKM) RNA-seq data were used for the following analysis. The scRNA-seq dataset of HCC was downloaded from the GEO dataset (GSE149614). The gene expression matrix was used for further analysis. A total of 3,012 phosphorylation-related genes (PRGs) were extracted from the Gene Ontology database (GO) (Supplementary Table S1).

### Differentially Expressed Gene (DEG) Analysis and GO Enrichment Analysis

DEGs were analyzed by analysis of variance (ANOVA) with the cutoff of FDR<0.01 and fold change>2. GO enrichment analysis was performed by DAVID (https://david.ncifcrf.gov/). The results were visualized with the ggplot2 R package (*p* < 0.01) (Law et al., 2014).

### Prognosis-Related Genes Analysis

Univariate Cox regression analysis was used to identify differentially expressed genes associated with overall survival in HCC patients from the TCGA dataset. A risk score was calculated for each patient, a median value was identified for all patients, and HCC patients were then divided into low-risk (score below the median) and high-risk (score above the median) groups. The high- and low-risk groups were stratified and visualized using Kaplan–Meier (K-M) survival curves and analyzed for statistical significance using the log-rank test. Cox regression analysis and Kaplan–Meier curves with the log-rank test were conducted by the glmnet and survival packages.

### Gene Expression, Survival, Tumor Grade, and Nodal Metastasis Status Analysis of HCC

The boxplot of gene candidates was performed by GEPIA2 (http://gepia2.cancer-pku.cn/), which includes the gene expression data of TCGA and GTEX. The survival plot was generated by the survival module of GEPIA. Tumor grade, nodal metastasis status, and *TP53*-mutant status analyses were performed using the UALCAN database (http://ualcan.path.uab.edu/). The protein expression of AURKA and EZH2 in HCC was explored based on immunohistochemistry (IHC) data from the Human Protein Atlas (HPA) database (http://www.proteinatl.as.org/).

### ScRNA-Seq Analysis

Basic filtering, classification, and visualization of the single-cell dataset of HCC were analyzed with the Seurat package (v.3.0) in R (v.3.4.0.5). We trimmed cells expressing fewer than 200 unique genes, more than 4,500 unique genes or over 20% mitochondrial reads. The top 2000 variable genes were used for further clustering. The function FindMarkers was used based on *t*-test. Fifteen principal components (PCs) remained for t-SNE analysis. Cell types were identified by the markers compared with information in the CellMarker database (http://biocc.hrbmu.edu.cn/CellMarker/index.jsp) (Satija et al., 2015; Zhang et al., 2019b). Single-cell RNA-seq analysis of ovarian carcinoma was performed by the CancerSCEM database (Cancer Single-cell Expression Map database) (Zeng et al., 2021). We applied the E-MTAB-8559 dataset for further analysis (Nelson et al., 2020).

### Gene Expression Correlation Analysis and Cell Cycle Status Inference Analysis

Gene expression correlation analysis was performed by the Pearson method in R (4.0.5) (Pearson coefficient >0.2, *p* value <0.001). Cell cycle status inference was based on the cyclone method in the scran package in R (4.0.5) (Haghverdi et al., 2018).

### QIAGEN Ingenuity Pathway Analysis

IPA was used to analyze the potential regulatory genes and pathways of the phosphorylation-related genes. The related diseases and biofunctions were analyzed by the disease and function module of IPA.

### Cell Culture and Drugs

Human HCC cell lines (Hep3B and Huh7) were purchased from Beijing Cell Resource Center, Institute of Basic Medical Sciences, Chinese Academy of Medical Sciences. Cells were cultured in Dulbecco’s Modified Eagle Medium (DMEM, HyClone, United States, SH30243-01) with 10% fetal bovine serum (FBS, HyClone, United States, SH30084) and 1% penicillin/streptomycin (HyClone, United States, SV30010) at 37°C in 5% CO2. The EZH2 inhibitor gambogenic acid was purchased from MCE (Shanghai, China). The AURKA inhibitor alisertib was purchased from SELLECK (Shanghai, China). The solvent for gambogenic acid and alisertib is DMSO (Sigma Aldrich, United States).

### Cell Viability Assays

Cell viability was assessed using Calcein-AM/PI staining assays (Beyotime, Shanghai, C2015M). DAPI (Invitrogen, Carlsbad, CA) was used to stain the cell nuclei. A total of 1×10^6^ Hep3B or Huh7 cells were plated into 96-well plates for 24 h to allow for cell attachment before being incubated for an additional 48 h with various concentrations of the tested compounds. The concentrations of gambogenic acid or DMSO (solvent of gambogenic acid) were 0 μM (Ctrl), 0.1, 2, 10, 50, and 100 μM. The concentrations of alisertib or DMSO (solvent of alisertib) were 0 μM (Ctrl), 0.1, 1, 10, 50, and 100 μM. For slope analysis, a total of 1×10^6^ Hep3B or Huh7 cells were plated into 96-well plates for 24 h to allow for cell attachment before being incubated for additional time (0, 12, 24, 36, 48, and 60 h) with gambogenic acid (2 μM) and alisertib (10 μM), respectively. The cells were then cultured with Calcein-AM, PI and DAPI at 37°C for 30 min according to the manufacturer’s protocol. Subsequently, images were captured using a PerkinElmer Operetta CLS High Content Screening System. All assays were conducted at least three times.

### Colony Formation Assay

Hep3B and Huh7 cells (a total of 5×10^6^) were plated into 6-well plates, respectively. After treatment with an EZH2 inhibitor (2 μM gambogenic) or an AURKA inhibitor (10 μM alisertib) for 72 h, colonies were fixed with 4% paraformaldehyde (Mei Lun, China, MA0192) and stained with crystal violet (Beyotime, Shanghai, C0121-100 ML) for 30 min at room temperature. The visible colonies were counted manually. All assays were conducted at least three times.

### Wound-Healing Assay

A total of 5×10^6^ Hep3B and Huh7 cells were seeded into 6-well plates and grown to 80% cell abundance. Then, a single layer wound was created using a pipette tip, and we took images (Olympus, BX51). After treatment with an EZH2 inhibitor (2 μM gambogenic) or AURKA inhibitor (10 μM alisertib) for 24 h, imaging was repeated at the same location and further analyzed by ImageJ software. All assays were conducted at least three times.

### Transwell Assay

The capacity of cell migration and invasion was evaluated via Transwell (JET BIOFIL, Guangzhou, TCS004024) assays. The upper chamber was precoated with Matrigel (Corning, United States, 356234) for the invasion assay, whereas the migration assay was not precoated with Matrigel. A total of 5×10^6^ Hep3B and Huh7 cells were resuspended in serum-free DMEM and placed in the upper chamber of the Transwell system. After culturing overnight, 10% FBS was used as a chemoattractant and placed in the lower chamber. The control (1XPBS), EZH2 inhibitor (2 μM gambogenic), or AURKA inhibitor (10 μM alisertib) were added to the top chamber. After culturing for 24 h, the chambers were fixed with 4% paraformaldehyde and stained with crystal violet (Beyotime, Shanghai, C0121-100 ml) for 30 min at room temperature. After removing the noninvasive cells, five fields in the chamber were photographed using an optical microscope (Olympus, BX51), and the numbers of cells were counted. All Transwell assays were conducted at least three times.

### Statistical Analyses

Data were expressed as means ± s.d. For all experiments were analyzed by Student’s t-test. Differences were considered statistically significant if *p* < 0.05. *p < 0.05, **p < 0.01, ***p < 0.001. The statistical analyses were conducted in R 3.4.0.5. The dendrogram was computed and visualized using the R package ggplot2.

## Results

### Aberrant Phosphorylation Processes Were Related to Hepatocellular Carcinoma Progression

**FIGURE 1:**
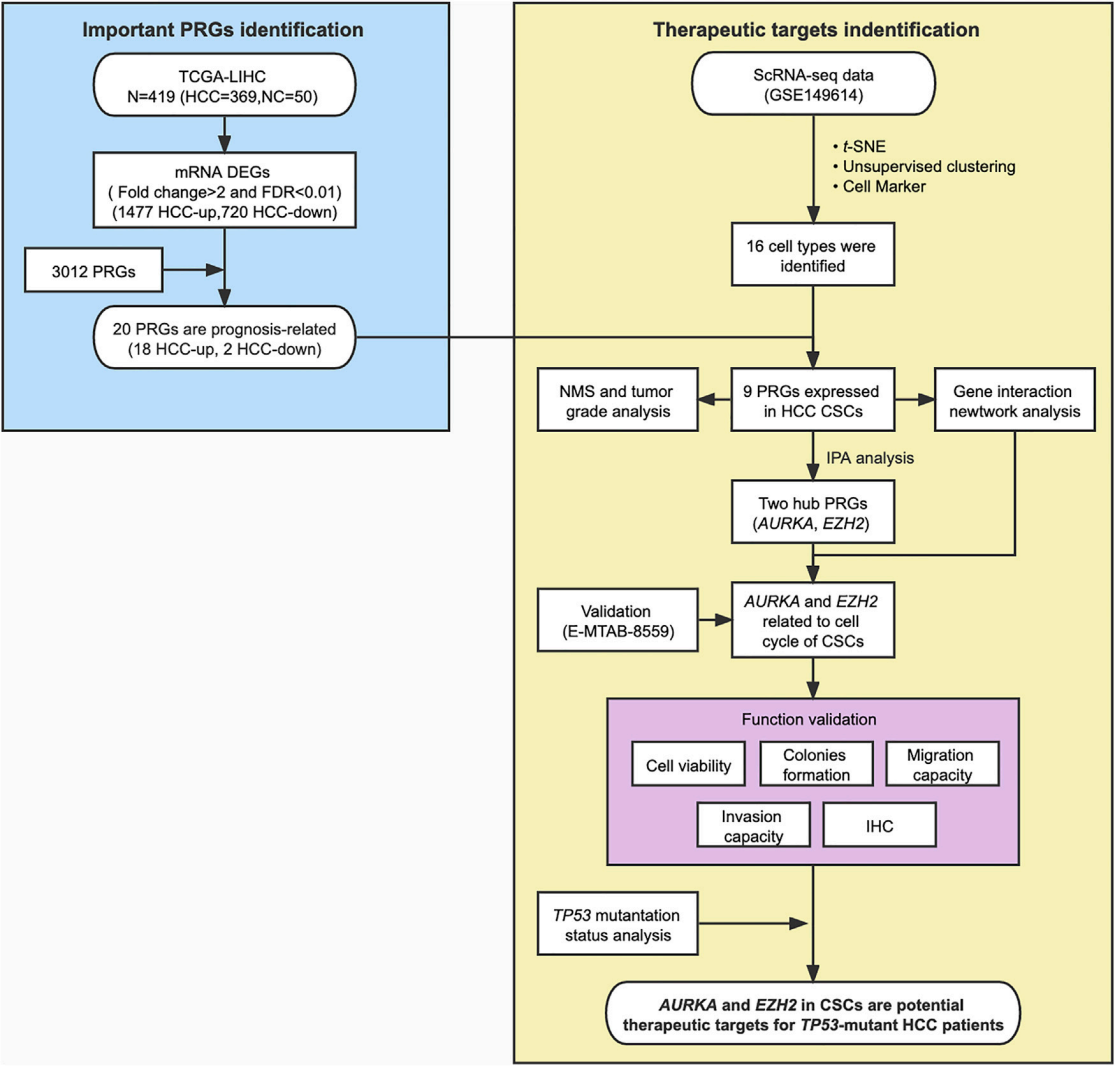
Flowchart for bioinformatics analysis and validation of phosphorylation-related genes (PRGs) based on the TCGA dataset and scRNA-seq data of HCC. NMS, Nodal Metastasis Status; DEGs, differentially expressed genes.

**FIGURE 2:**
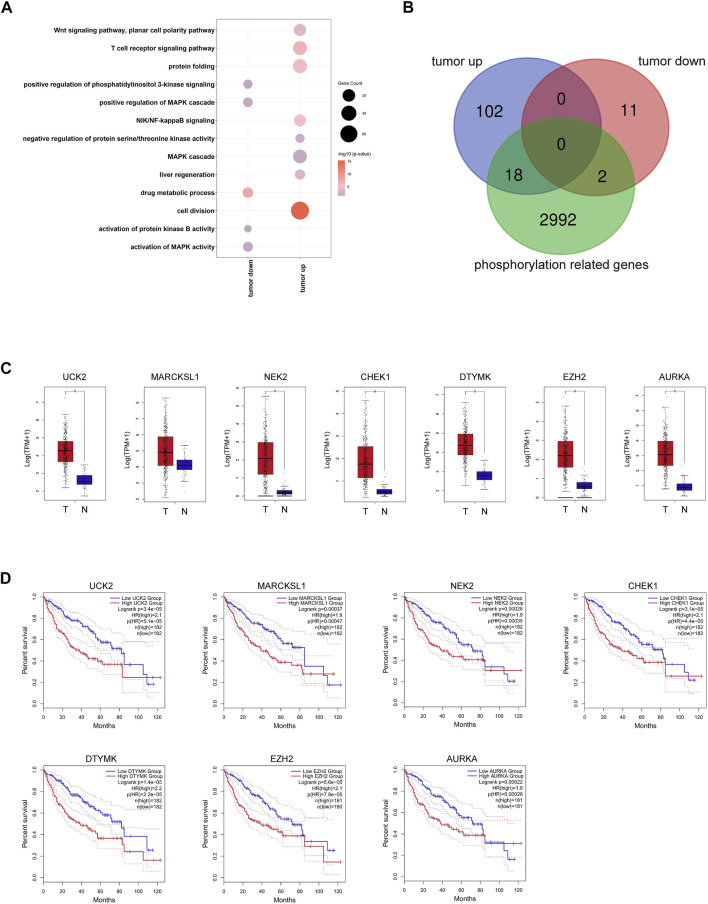
Transcriptome profiling of PRGs in the TCGA LIHC dataset. **(A)** Representative GO terms of HCC related DEGs. Dot size represents the gene counts, and dot color represents the significance. **(B)** The overlay of 120 prognosis-related and HCC upregulated DEGs, 13 prognosis-related and HCC downregulated DEGs, and 3,012 kinase-related genes. **(C)** Expression of seven representative PRGs. **(D)** Kaplan–Meier overall survival curves of TCGA LIHC patients grouped by seven representative PRGs.

We showed the workflow chart of this study in Figure 1. To identify the key genes involved in the occurrence and progression of hepatocellular carcinoma (HCC) as well as prognosis, we conducted an in-depth analysis of public RNA-Seq data from the TCGA database of 369 HCC and 50 adjacent normal tissues. According to the results of differentially expressed gene (DEG) analysis, we identified 1,477 upregulated and 720 downregulated genes in HCC tissues compared with adjacent normal tissues (fold change >2, FDR <0.01) (Supplementary Table S2). Interestingly, after gene ontology (GO) enrichment by DAVID, we found that the upregulated genes were not only enriched in cell division, the T cell receptor signaling pathway, and other particular pathways that have been verified during tumorigenesis but also enriched in some phosphorylation-related GO terms, including negative regulation of protein serine/threonine kinase activity, regulation of cyclin-dependent protein serine/threonine kinase activity and MAPK cascade pathways (Figure 2A, Supplementary Table S3). The downregulated genes were highly associated with activation of protein kinase B activity and positive regulation of phosphatidylinositol 3-kinase signaling pathways (Figure 2A, Supplementary Table S3). In HCC, those DEGs enriched in protein phosphorylation pathways indicated that protein phosphorylation might play an important role during HCC tumorigenesis. Then, the top 500 most prognosis-related genes were selected by Cox regression analysis, among which 120 and 13 genes were significantly up- and downregulated in cancerous tissues (with the cutoff of fold change >2 and FDR<0.01), respectively (Figure 2B, Supplementary Table S4). Furthermore, filtered by the Gene Ontology database, we identified 20 DEGs that were associated with prognosis and were closely related to the activity and function of protein kinases (Figure 2B, Supplementary Table S5). Among them, the expression levels of the above 18 phosphorylation-related genes (PRGs) were significantly upregulated in HCC samples (Figure 2C, Supplementary Figure S1A), while the 2 phosphorylation-related genes were significantly downregulated in HCC samples (Supplementary Figure S1A). The survival curves illustrated that the patients with the higher expression levels of HCC-upregulated and PRGs showed significantly poor prognosis (Figures 2D, Supplementary Figure S1B). Among them, *UCK2*, *MARCKSL1*, *NEK2*, *CHEK2*, *AURKA*, and *DTYMK* are protein kinases, and others, such as *EZH2* and *PRC1*, are PRGs. Additionally, the two HCC-downregulated genes (*SLC11A1* and *ADRA2B*) showed significantly downregulated in HCC samples. The high expression of *SLC11A1* and *ADRA2B* was associated with poor and good prognosis, respectively, due to the diverse functions of phosphorylation (Supplementary Figure S1B). The abnormal expression of these genes might induce the dysregulation of the phosphorylation of HCC progression-related genes and enhance tumorigenesis and tumor development. We suspected that these important genes may participate in protein phosphorylation, affecting downstream phosphorylation-related pathways and then promoting the occurrence and progression of HCC.

### ScRNA-Seq Dataset Indicates the High Expression of PRGs in Cancer Stem Cells

**FIGURE 3:**
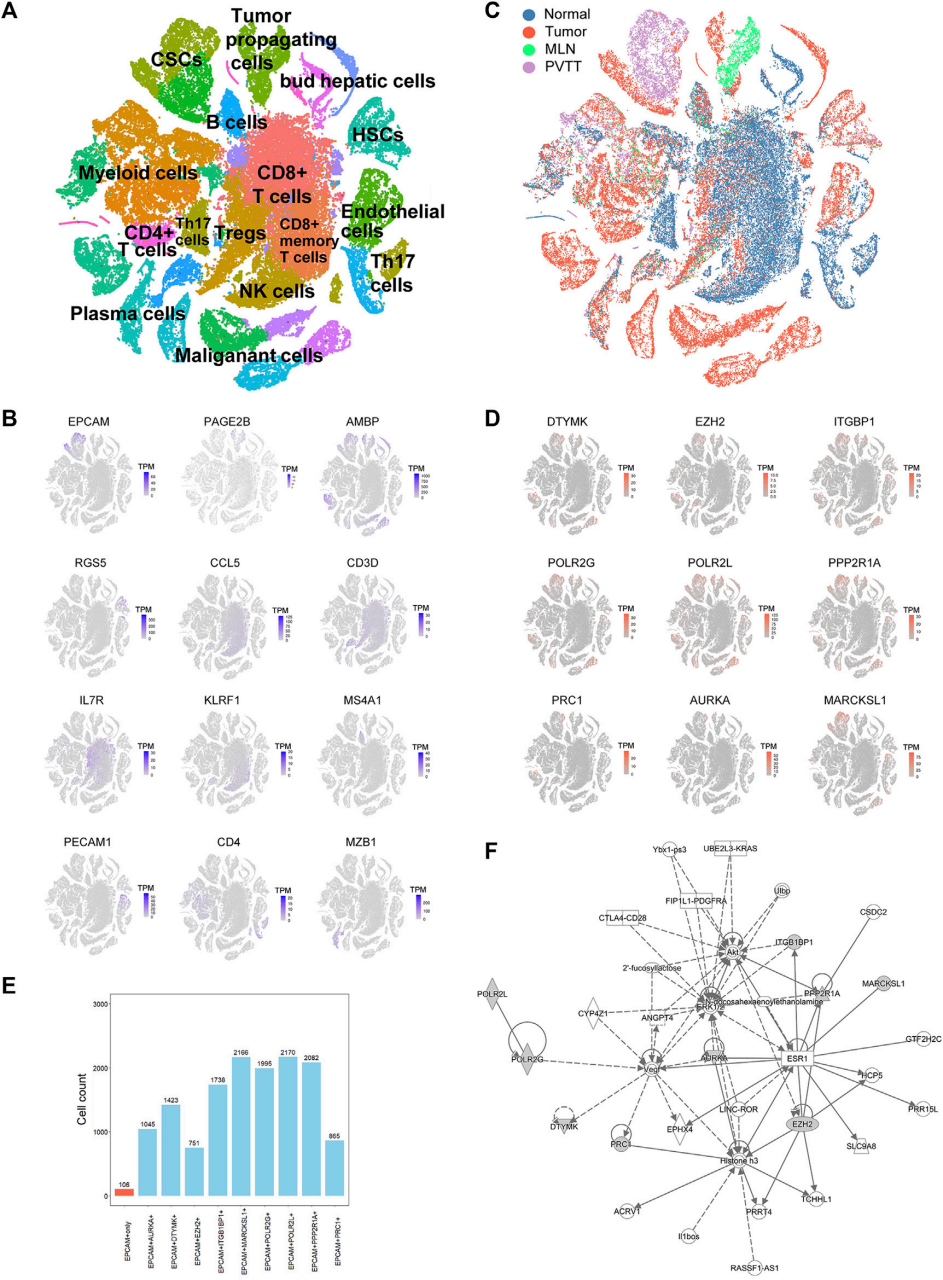
Single-cell RNA sequencing (ScRNA-seq) analysis of HCC patients. **(A)** t-SNE clustering of scRNA-seq colored by significant cell types. **(B)** t-SNE plot of expression for genes specifically upregulated in each of the clusters. **(C)** t-SNE clustering of scRNA-seq as in **(A)** but colored by patient type. Normal: nontumor liver, Tumor: primary tumor, MLN: metastatic lymph node, PVTT: portal vein tumor thrombus. **(D)** t-SNE plot of expression for nine PRGs that were significantly highly expressed in tumor-related cell clusters. **(E)** Statistics of the cell counts of the nine PRGs coexpressed with EPCAM in 2,795 cancer stem cells (CSCs). **(F)** Regulatory network analysis of the nine PRGs using Ingenuity Pathway Analysis (IPA).

Traditional RNA-Seq can only detect transcriptome information of the entire tumor tissue with the limitations that it cannot distinguish different cell types, such as CSCs, malignant cells, and immune cells. To confirm which exact cell type the above-mentioned genes were affected, we downloaded the scRNA-seq data of HCC, containing the primary tumor, portal vein tumor thrombus (PVTT), metastatic lymph node, and nontumor liver controls of 10 patients from the GEO database (GSE149614). After quality filtering using the Seurat package, approximately 70,000 single cells were included in further analysis (Supplementary Figure S2A). The top 2000 variable genes were used for further clustering (Supplementary Figure S2B). Fifteen principal components (PCs) remained for t-SNE analysis (Supplementary Figure S2C). We identified 12 major cell types, including CSCs, tumor propagating cells, myeloid cells, CD4^+^ T cells, CD8^+^ T cells, Treg cells, HSCs, B cells, NK cells, plasma cells, malignant cells, and endothelial cells, which were labeled with canonical markers (Figures 3A,B, Supplementary Figure S3). These cells were mainly tumor and immune cells, some of which served as tumor-specific cell subgroups (Figure 3C). Since all of the 18 HCC-upregulated and PRGs were consistently related to poor prognosis, to investigate their potential locations in HCC, we used the scRNA-seq data and found that 18 HCC-upregulated and PRGs were mostly expressed in tumor-related cells but not in immune cells. Of all the tumor related cells, such as malignant cells and bud hepatic cells, we detected the expression of some of the PRGs to some extent. However, CSCs caught our attention due to its high expression levels of the PRGs and crucial functions in tumor progression. Considering the sample heterogeneity, we identified a total of nine (*DTYMK*, *EZH2*, *ITGBP1*, *POLR2G*, *POLR2L*, *PPP2R1A*, *PRC1*, *AURKA*, and *MARCKSL1*) genes that were significantly highly expressed in CSCs (Figure 3D). Seven of the remaining genes (*UCK2*, *CCNB1*, *CDC25C*, *CDK1*, *CDKN2C, CHEK1, and NEK2*) were expressed at low levels in CSCs, and two of the remaining genes were not expressed in CSCs (*BAMBI*, *STK39*) (Supplementary Figure S4A). We further examined their expression in HCC CSCs. Interestingly, of all 2795 *EPCAM* + liver CSCs, most expressed at least one of the nine genes, while only 106 cells expressed *EPCAM* only, indicating the crucial roles of the nine PRGs in liver CSCs (Figure 3E, Supplementary Table S6). Among them, *POLR2L*, *MARCKSL1*, *PPP2R1A*, *POLR2G*, and *ITGB1BP1* showed the highest expression levels in HCC CSCs, indicating that these five genes may participate in phosphorylation-related pathways in HCC CSCs (Supplementary Figure S4B). Furthermore, as the overlapping relationship of the five genes and CSC marker *EPCAM* showed in the Venn diagram, most cells expressed two or more PRGs, while cells expressing both *POLR2L* and *MARCKSL1* accounted for the highest proportion (Supplementary Figure S4B). To further investigate the potential biological mechanisms and functions of the key genes we identified, QIAGEN Ingenuity Pathway Analysis (IPA) was used to analyze the potential regulatory genes and pathways of the nine PRGs. The results showed that they might be involved in regulating the estrogen receptor 1 (ESR1), vascular endothelial growth factor A (VEGA), and MAP kinase (ERK1/2) pathways (Figure 3F). The hub genes in this IPA network were *AURKA* and *EZH2*, which might exercise a core influence on phosphorylation-dependent pathways in HCC. In addition, the most related disease of those genes was presumed to be cancer-related (Supplementary Figure S4C, Supplementary Table S7). The above results illustrated the possible important functions and potential regulatory mechanisms of PRGs in CSCs.

### Expression of PRGs Is Related to Tumor Grade and Nodal Metastasis Status

**FIGURE 4:**
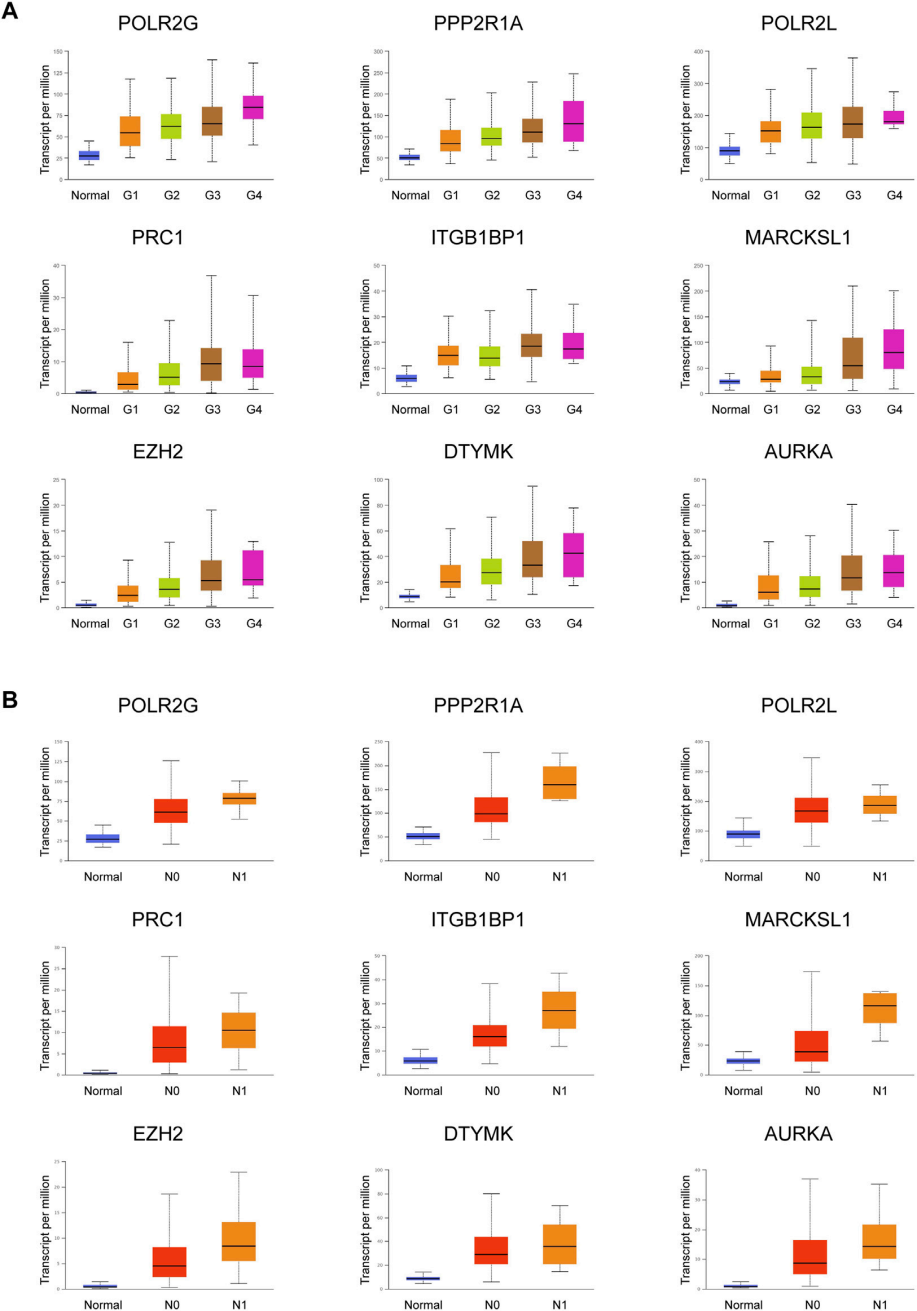
Expression of nine important PRGs is related to tumor grade and nodal metastasis status. **(A)** Expression of the nine PRGs at different tumor grades in HCC patients (n_Normal = 50, n_Grade 1 = 54, n_Grade 2 = 173, n_Grade 3 = 118, and n_Grade 4 = 12). **(B)** Expression of the nine PRGs at different nodal metastasis statuses in HCC patients (n_Normal = 50, n_N0 = 252, n_N1 = 4). The statistical significance of the relationship between genes and tumor grade/nodal metastasis status was shown in Supplementary Table S8.

Important PRGs may regulate the occurrence and progression of tumors by affecting the activity of CSCs. Tumor progression can be defined by tumor grade and nodal metastasis status (NMS). Tumor grade was defined as normal, G1, G2, G3, and G4. The lower the tumor grade, the better the tumor differentiation. Nodal metastasis statuses N0 and N1 indicate no regional lymph node metastasis and 1 to 3 axillary lymph nodes, respectively. The clinical information of the TCGA LIHC patients included in this study was shown in Table 1. Among the highly expressed important PRGs, the increased expression levels of *POLR2G*, *PPP2R1A*, *POLR2L*, *PRC1*, *ITGBP1*, *MARCKSL1*, *EZH2*, *DTYMK*, and *AURKA* were accompanied by increased tumor grade (Figure 4A, Supplementary Table S8), which indicated that the RNA abundance of these genes was positively correlated with higher tumor grade and lower tumor differentiation level. Furthermore, the higher the nodal metastasis status, the higher the expression levels of *POLR2G*, *PPP2R1A*, *POLR2L*, *PRC1*, *ITGB1BP1*, *MARCKSL1*, *EZH2*, *DTYMK*, and *AURKA* (Figure 4B, Supplementary Table S8), which illustrated the potential relationship of these genes and nodal metastasis. The results demonstrated a positive correlation between PRGs and tumor grade as well as nodal metastasis status, which were associated with tumor progression.

**TABLE 1:** Clinical characteristics of TCGA LIHC patients included in this study.

Characteristics	Number of patients
Grage	
Normal	50
Grade1	54
Grade2	173
Grade3	118
Grade4	12
Nodal metastasis status	
Normal	50
N0	252
N1	4
*TP53* mutation	
Normal	50
*TP53*-Mutant	105
*TP53*-NonMutant	255

### PRGs Participate in the Cell Cycle Transition of HCC CSCs

**FIGURE 5:**
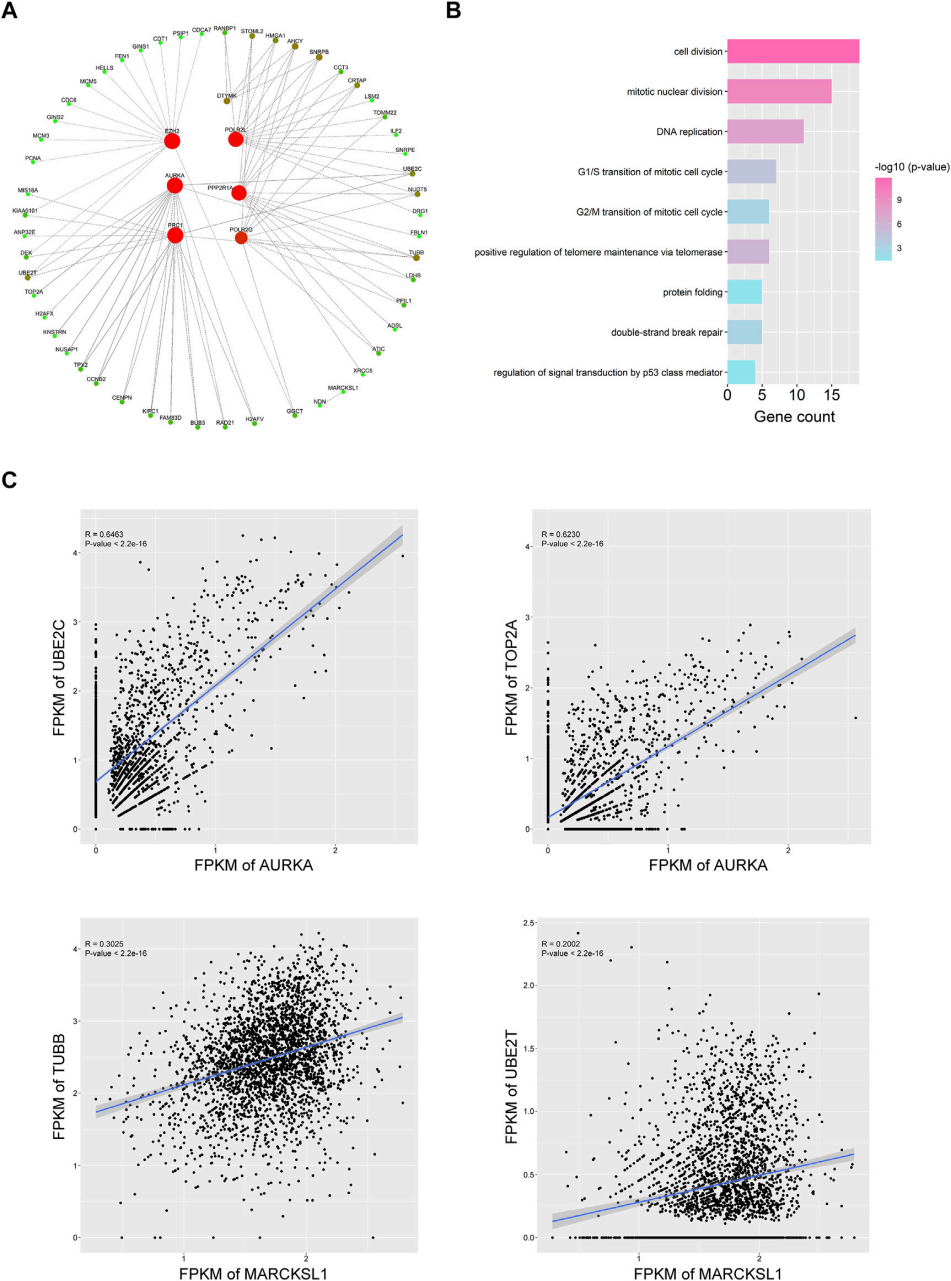
Nine important PRGs participate in the cell cycle transition of HCC CSCs. **(A)** Coexpression network of the nine PRGs and their representative correlated genes in HCC CSCs. Interactions with coefficient >0.3 and *p* value < 0.001 were selected for plotting. **(B)** Representative GO terms of correlated genes in **(A)**. **(C)** Typical examples of coexpression of PRGs and cell cycle-related genes in HCC CSCs.

To explore the possible mechanism of how phosphorylation-related genes affect HCC progression, we predicted the co-expressed genes of each important PRGs, identified the genes with a Pearson correlation coefficient >0.2 (*p* < 0.001), and further constructed a possible gene expression regulatory network for HCC CSCs (Figure 5A, Supplementary Table S9). We found that different PRGs shared many related genes in the network, and these genes were enriched in cell division, mitotic nuclear division, and DNA replication (Figure 5B), indicating that PRGs might mediate multiple biological processes of tumor stem cells by regulating the expression of cell cycle-related genes such as *TOP2A*, *UBE2C*, and *CENPN* (Figure 5A). Additionally, there were strong correlations between the expression levels of *UBE2C*, *TOP2A*, and *AURKA* and between the expression levels of *TUBB*, *UBE2T*, and *MARCKSL1,* suggesting that *UBE2C* and *TOP2A* and *TUBB* and *UBE2T* may serve as potential targets for *AURKA* and in regulating the cell cycle (Figure 5C, Supplementary Table S9). The above results showed that the PRGs we identified, such as *AURKA*, *MARCKSL1*, and *EZH2,* might mediate the occurrence and progression of HCC by regulating cell cycle-related pathways in liver cancer stem cells. To investigate the effect of PRGs on the cell cycle, we used the cyclone function of the R package scran to infer the cell cycle status of CSCs based on the expression levels of different cell cycle genes. Interestingly, after correlation analysis, which was performed between the expression level of phosphorylation-related genes and the cell cycle inference score of each cell in the G1, S, and G2/M phases, we found that the correlation coefficient with S phase and G2/M phase was significantly higher than that of G1 phase, indicating the potential roles of PRGs in the activation of cell cycle checkpoints (Supplementary Figure S5A). The expression levels of *PRC1*, *AURKA*, *DTYMK*, and *EZH2* had the most significant correlation with the S phase and G2/M phase scores (Supplementary Figure S5A).

Among all these PRGs, *AURKA* and *EZH2* drew our attention because these two genes were hub genes in the network sourced from IPA (Figure 3F) and were previously considered functional mostly in tumor progression. To further confirm the roles of *AURKA* and *EZH2* in the cell cycle pathway of cancer stem cells, we analyzed single-cell RNA-seq datasets of ovarian carcinoma in the CancerSCEM database (Cancer Single-cell Expression Map database) based on the E-MTAB-8559 dataset (Wu et al., 2016; Davient et al., 2018; Doan et al., 2019). We found that *AURKA* and *EZH2* were mostly expressed in cells expressing *EPCAM*, which is consistent with the results of GSE149614 (Figure 3, Supplementary Figure S5B). The cell cycle scores of in G2/M and S phases of EPCAM^+^ cells were positively correlated with the expression of *AURKA* and *EZH2* (Supplementary Figure S5C). These results indicated that PRGs such as *AURKA* and *EZH2* might participate in cell cycle-related pathways and then mediate the proliferation of tumor cells, especially CSCs.

### Gambogenic Acid (EZH2 Inhibitor) and Alisertib (AURKA Inhibitor) Inhibit HCC Cell Proliferation, Migration, and Invasion

**FIGURE 6:**
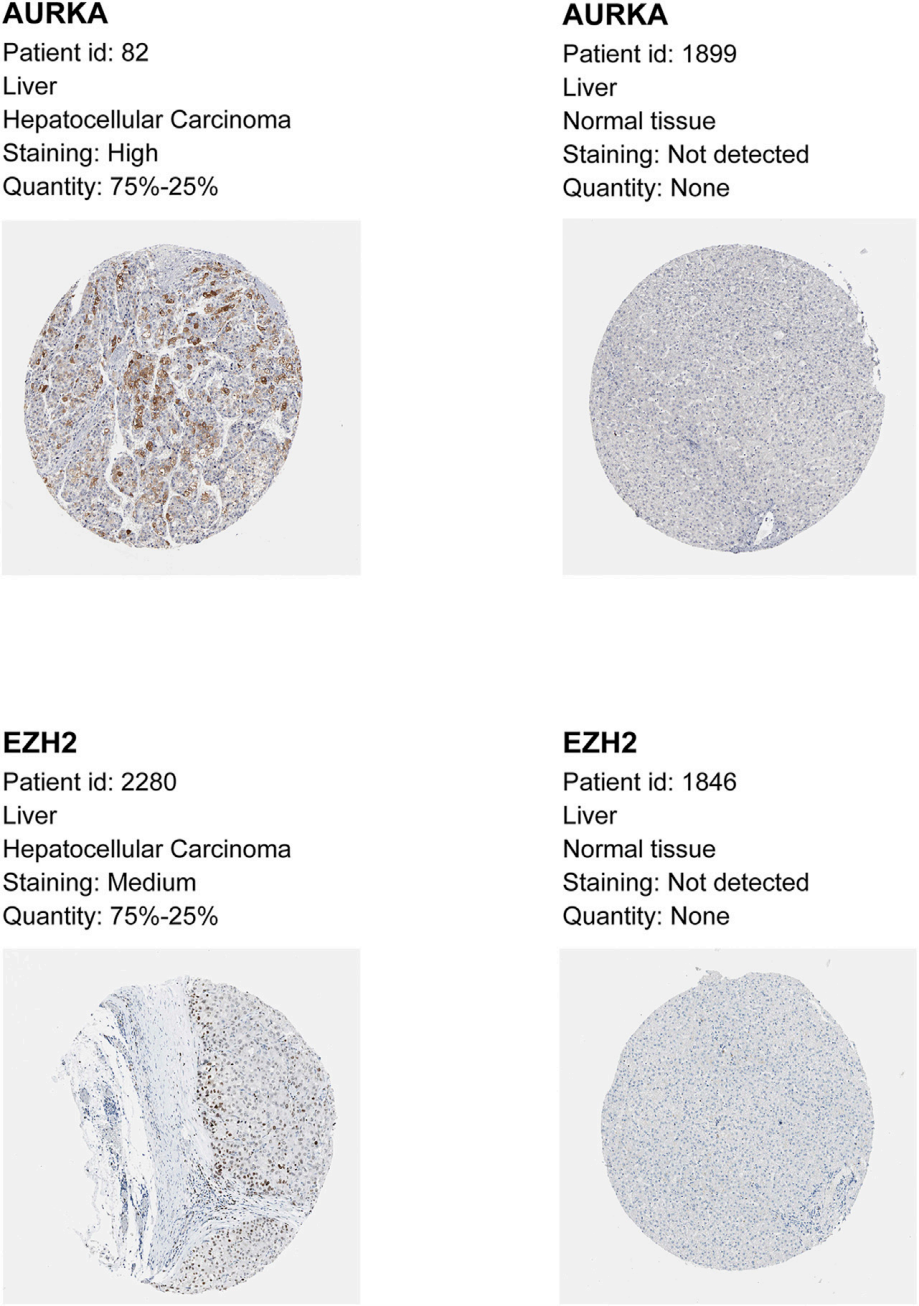
Immunohistochemistry (IHC) data of AURKA and EZH2 from the Human Protein Atlas. Higher expression of AURKA and EZH2 by immunohistochemistry in HCC compared with normal tissue.

**FIGURE 7:**
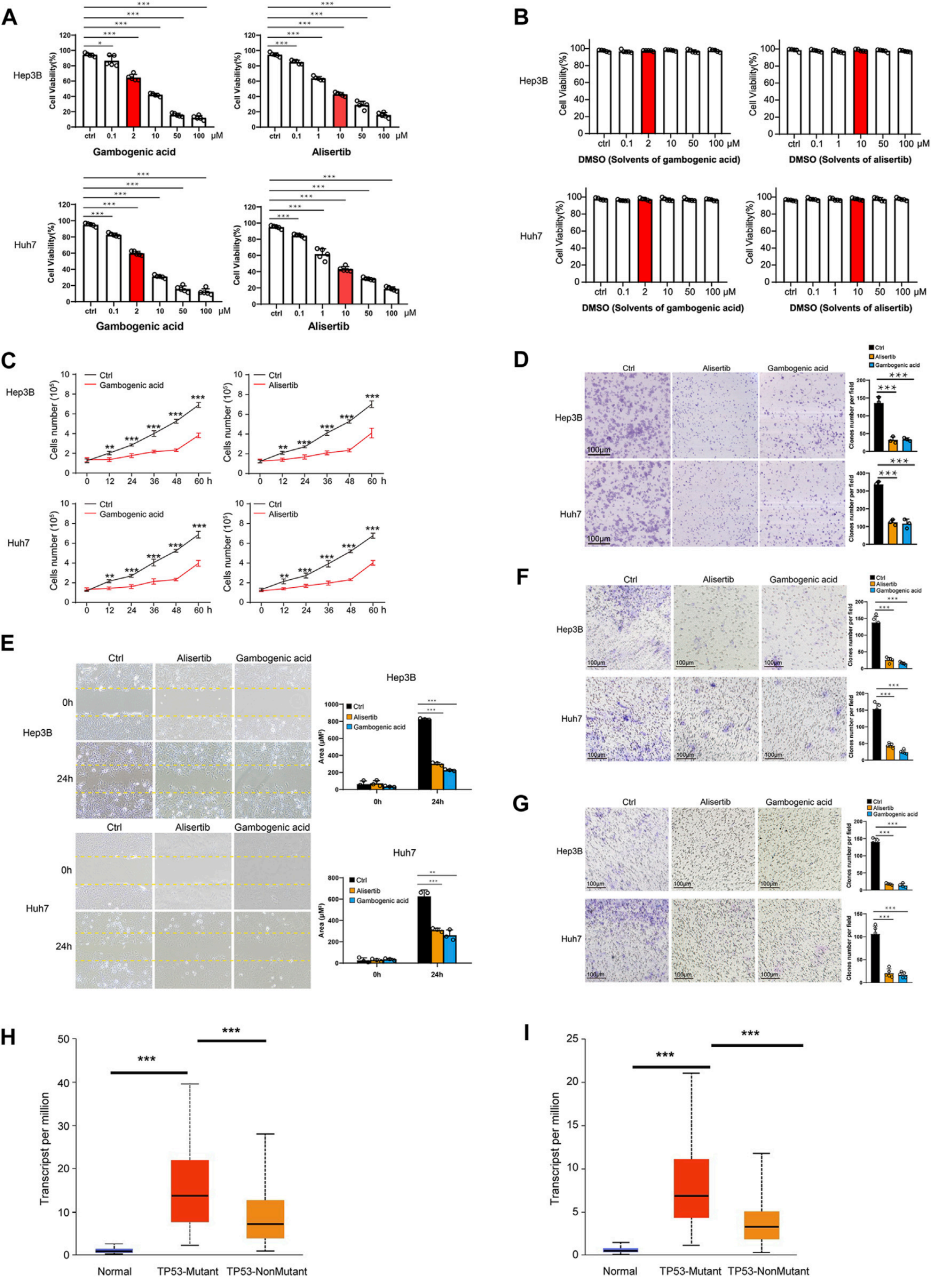
Gambogenic acid (EZH2 inhibitor) and alisertib (AURKA inhibitor) inhibit HCC cell proliferation, migration, and invasion. **(A)** Hep3B and Huh7 cells were treatedwith gambogenic acid (EZH2 inhibitor) or alisertib (AURKA inhibitor) at different concentrations (0–100 μM) for 48 h, and cell viability was determined by Calcein–AM/PI staining assays. **(B)** Hep3B and Huh7 cells were treatedwith DMSO(solvents of gambogenic acid and alisertib) at different concentrations (0–100 μM) for 48 h, and cell viability was determined by Calcein–AM/PI staining assays. **(C)** Hep3B and Huh7 cells were treated with gambogenic acid (EZH2 inhibitor, 2 μM) or alisertib (AURKA inhibitor, 10 μM) for 0, 12, 24, 32, 48, and 60 h, and cell viability was determined by Calcein–AM/PI staining assays. **(D)** Colony formation assays were conducted to analyze Hep3B and Huh7 cell proliferation with gambogenic acid (2 μM) or alisertib (10 μM) treatment. **(E, F)**Wound healing assays **(E)** and Transwell assays **(F)** were performed to detect the cellmigratory abilities of Hep3B and Huh7 cells treated with gambogenic acid (2 μM) or alisertib (10 μM). **(G)** Transwell assays were performed to detect the cell invasion abilities of Hep3B and Huh7 cells treatedwith gambogenic acid (2 μM) or alisertib (10 μM). Data are expressed as themeans ± s.d. Differences were considered statistically significant if *p* < 0.05. ns, no significance, *p < 0.05, **p < 0.01, ***p < 0.001. **(H, I)** Expression of AURKA **(H)** and EZH2 **(I)** in TCGA–LIHC based on TP3 mutation status.

To verify the protein levels of AURKA and EZH2 in HCC between HCC tissues and normal tissues, we applied the IHC data in the HPA database and found that the expression of AURKA and EZH2 was significantly higher in HCC tissue than in normal tissues (Figure 6). To study the functions of *EZH2* and *AURKA* in HCC, we treated HCC cell lines, including Hep3B and Huh7 cells, with an EZH2 inhibitor (gambogenic acid) and an AURKA inhibitor (alisertib) *in vitro*. The Calcein-AM/PI staining results showed that the EZH2 inhibitor (2 μM gambogenic acid) and AURKA inhibitor (10 μM alisertib) significantly impeded the proliferation of Hep3B and Huh7 cells after 48 h treatment (Figure 7A). Since inhibitors’ concentration was increased gradually, then the corresponding solvent (DMSO) treatments were used as controls. The results proved that the observed effect of inhibitor was independent of the solvent (Figure 7B). Because of a high dose of inhibitors and or solvent can induce apoptosis, cell proliferation rate (slope analysis) was examined to prove that inhibitors impede cell proliferation at 48 h treatment (Figure 7C). These results were consistent with previous reports in breast cancer, nasopharyngeal carcinoma, and lung cancer (Korobeynikov et al., 2019; Yan et al., 2011; Yu et al., 2012). In addition, the number of colonies formed (Figure 7D), migration (Figures 7E,F), and invasion capacities (Figure 7G) were also significantly decreased in Hep3B and Huh7 cells treated with EZH2 and AURKA inhibitors. Notably, *TP53*-mutated or *TP53*-deleted human HCCs were more hypersensitive to the treatment of AURKA inhibitors in previous studies (Dauch et al., 2016; Caruso et al., 2019). We found that both *AURKA* and *EZH2* were highly expressed in *TP53*-mutant HCC samples (Figures 7H,I), suggesting the relationship of *AURKA* and *EZH2* with *TP53* mutation. These results showed that *EZH2* and *AURKA* play an important role in promoting the proliferation, migration, and invasion of HCC, especially in *TP53*-mutant HCC, which indicates that they are potential targets for clinical therapy.

## Discussion

In this study, we provided a comprehensive understanding of protein kinases, phosphatases, and other phosphorylation-related genes (PRGs) at both the bulk RNA and single-cell RNA levels in HCC. We investigated their expression levels using TCGA datasets and scRNA-seq data from GEO datasets. Notably, we found that PRGs play crucial roles in liver cancer stem cells. We further validated two essential inhibitors of PRGs (*AURKA* and *EZH2*) in HCC cell lines, which suppressed cell proliferation, clone formation, migration, and invasion capacities.

To our knowledge, the PRGs in CSCs of HCC remain largely unknown. The application of scRNA-seq methods has enabled us to build precise profiling of multiple cell types in HCC, such as CSCs, cancer-associated structural cells, vascular cells, fibroblasts, and immune cells, and illustrate the subset of cell types that promote tumor progression and metastasis. Previous studies have already shown the heterogeneity of immune cells in HCC by scRNA-seq technology (Zheng et al., 2017; Zhang et al., 2019a). In this study, we focused on the PRGs of CSCs based on scRNA-seq data of 10 HCC patients from the GEO database (GSE149614). The results showed that PRGs tend to be expressed abnormally in CSCs instead of immune cells, indicating their potential roles in CSC proliferation, differentiation, and migration.

Due to the heterogeneity of individual HCC patients, only nine genes were detected in the scRNA-seq dataset among the 18 important PRGs identified in the TCGA LIHC dataset. This result illustrated the conservation of the nine genes in HCC patients. Among the nine PRGs, four of them (*EZH2*, *POLR2G*, *POLR2L*, and *PRC1*) also regulate gene expression through other pathways, such as histone methyltransferase and transcription regulation (Acker et al., 1996; Maeta et al., 2020; Xue et al., 2021). In our study, we found that the increased expression of those genes also correlated with higher progressive tumor grades and advanced metastatic stages. Among them, four genes (*PRC1*, *AURKA*, *DTYMK*, and *EZH2*) were calculated by cell cycle inference to be more correlated with the cell cycle. Since the hub genes in the IPA network were *AURKA* and *EZH2*, we further validated the role of *AURKA* and *EZH2* in tumor cell proliferation and migration in HCC cell lines. Our results indicate that an AURKA inhibitor (alisertib) and an EZH2 inhibitor (gambogenic) can obviously inhibit the proliferation, migration, and invasion of HCC cells, which are potential targets for clinical application.

*AURKA* plays an important role in mitosis, including centrosome function and maturation, spindle assembly, chromosome alignment, and mitotic entry (Bolanos-Garcia, 2005; Nikonova et al., 2013). Overexpression of *AURKA* correlates with tumor progression and poor prognosis in various carcinomas, including pancreatic carcinoma and breast carcinoma (Gomes-Filho et al., 2020). Furthermore, blockade of *AURKA* in preclinical models of ovarian carcinoma leads to decreased proliferation and increased apoptosis (Lin et al., 2008). Inhibition of AURKA affect the vasculogenic mimicry formation of CSCs in triple negative breast cancer (Sun et al., 2016). In HCC, the overexpression of *AURKA* has been reported to be related to aggressive tumor characteristics, chemotherapy resistance, and poor prognosis (Jeng et al., 2004; Lin et al., 2010; Zhang et al., 2014). Alisertib is a novel oral adenosine triphosphate-competitive AURKA inhibitor. A phase II study of alisertib in advanced sarcoma showed promising results for liposarcoma (PFS at 12 weeks of 73%), leiomyosarcoma (44%), and malignant peripheral nerve sheath tumors (60%), although each cohort only had a small number of patients (Dickson et al., 2016). Other studies have also shown the partial responses of alisertib in breast cancer, small-cell lung cancer, non-small-cell lung cancer, head and neck squamous cell carcinoma, and gastroesophageal adenocarcinoma (Melichar et al., 2015). *EZH2* is a catalytic subunit of polycomb repressive complex 2 (*PRC2*) (He et al., 2012). Based on H3K27me3-mediated gene expression silencing, *EZH2* often functions as a transcriptional repressor to downregulate tumor suppressors such as *ADRB2* and *DAB2IP* (Cao et al., 2002; Chen et al., 2005; Yu et al., 2007). Notably, the overexpression of EZH2 is reported to activate the phosphatidylinositol 3-kinase/Akt (PI3K/Akt) pathway, which is related to phosphorylation pathways (Gonzalez et al., 2011). Currently, *EZH2* is reported to be highly expressed in lymphomas, glioblastoma multiforme, ovarian, breast, and metastatic prostate cancers and is related to tumor progression, invasive growth, and poor prognosis in these tumors (Visser et al., 2001; Varambally et al., 2002; Bracken et al., 2003; Kleer et al., 2003; Yu et al., 2007; Hu et al., 2010; Orzan et al., 2011). Previous study has also revealed the activated EZH2 in glioma stem cells promoted cellular survival under stress and was potential to serve as tumor therapeutic targets (Jin et al., 2017). In addition, *EZH2* is reported to be related to proliferation and invasion in HCC cells (Liu et al., 2015; Zhang et al., 2017; Gao et al., 2020). Gambogenic acid (an *EZH2* inhibitor) is a natural compound derived from gamboge and is reported to be used as an antitumor drug in nasopharyngeal and lung cancer (Yan et al., 2011; Yu et al., 2012). Previous studies revealed that *AURKA* modulates the PI3K/Akt/mTOR pathway in Hep3B cells, and NICD1 and JAG1 were regulated by EZH2 (Zhu et al., 2017; Wang et al., 2020). In addition, *EZH2* could also affect tumor progression via histone methyltransferase-related functions. For example, *EZH2* is related to epigenetic silencing of miR-200c and induces BMI1-mediated hepatocarcinogenesis (Xu et al., 2020). *In vivo* experimental studies are needed to validate the functions of these two inhibitors in the progression of HCC in the future.

In this study, we found that *AURKA* and *EZH2* might mediate the occurrence and progression of HCC by regulating the transition of the cell cycle to S phase and G2/M phase in CSCs. We also found that both *AURKA* and *EZH2* were highly expressed in *TP53*-mutant HCC samples, suggesting the relationship of *AURKA* and *EZH2* with *TP53* mutation, corroborating a recent study in HCC cell lines and mice (Dauch et al., 2016; Caruso et al., 2019). These studies also showed that HCC cells with inactivating mutations in *TP53* were sensitive to alisertib. Additionally, other studies also reported the roles of *AURKA* and *EZH2* inhibitors.

The EZH2 inhibitor gambogenic acid can inhibit the growth of Hep3B and Huh7 cells through apoptotic pathways, and the inhibition of AURKA by alisertib leads to the inhibition of cell proliferation and induces cell cycle arrest and autophagy in Hep3B cells (Lee and Ho, 2013; Zhu et al., 2017). Thus, the AURKA inhibitor (alisertib) and the EZH2 inhibitor (gambogenic acid) may offer a potential therapeutic opportunity for HCC patients with *TP53* mutations, as *TP53* is the most frequently mutated tumor suppressor gene in HCC.

Taken together, the results of this study provide insights into the expression characteristics and potential functions of PRGs in HCC. The limitation of this study is that AURKA and EZH2 as key molecules in HCC cell proliferation were widely acknowledged. However, For the first time, we found AURKA and EZH2 were highly expressed in HCC CSCs that may contribute to the development of HCC. We found that they might play roles in the cell cycle transition of CSCs and validated that inhibitors of AURKA and EZH2 could suppress HCC proliferation and migration. Hence, alisertib and gambogenic acid have the potential to hold promise as novel anticancer agents of HCC, especially for *TP53*-mutant HCC. The therapeutic efficacy and mechanism of action of these compounds combined with other anticancer drugs are worth further clinical investigation as alternative combination therapies, which show promising potential in oncotherapy.

## Data Availability

The original contributions presented in the study are included in the article/Supplementary Material, further inquiries can be directed to the corresponding authors.

## Supplementary Material

Supplementary Figure S1 HCC prognosis-associated phosphorylation-related genes (PRGs). **(A)** Expression of 13 representative PRGs. **(B)** The Kaplan–Meier overall survival curves of TCGA LIHC patients grouped by the expression of 13 representative PRGs.

Supplementary Figure S2 Overview of single-cell RNA-seq (scRNA-seq) of HCC patients. **(A)** Quality control of scRNA-seq data. We filtered out cells with fewer than 200 unique genes, more than 4,500 unique genes, or over 20% mitochondrial reads. **(B)** The top 2,000 variable genes are shown by a characteristic variance diagram. **(C)** Data standard deviations under different selections of PCs.

Supplementary Figure S3 Top markers of different cell types of the scRNA-seq dataset (GSE149614). Top markers for CSCs are *EPCAM*, *MEG3*, *GLB1*, *VCX3A*, *VCX2*.

Supplementary Figure S4
**(A)** t-SNE plot of the expression of PRGs that were expressed at lower levels in CSCs. **(B)** Overlay of the CSCs expressing *EPCAM*, *POLR2L*, *MARCKSL1*, *POLR2G*, and *ITB1BP1*. **(C)** Treemap of diseases related to the nine PRGs.

Supplementary Figure S5 Gene expression and cell cycle scores of AURKA and EZH2 in scRNA-seq datasets. **(A)** Correlation between cell cycle score and expression of the nine PRGs in the GSE149614 dataset. **(B)** Gene expression of EPCAM, AURKA, and EZH2 in the single cells of the E-MTAB-8559 dataset. **(C)** Correlation between cell cycle score and expression of AURKA and EZH2 in the E-MTAB-8559 dataset.

## Glossary

PRGs: phosphorylation-related genes
FPKM: Fragments per kilobase million
GEO: Gene Expression Omnibus
GO: Gene ontology
ANOVA: analysis of variance
PC: prinicipal components
HCC: hepatocellular carcinoma
TKI: tyrosine kinase inhibitor
TCGA: the cancer genome atlas
CSC: cancer stem cell
AURKA: aurora kinase A
EZH2: enhancer of zeste homolog 2
DAVID: database for annotation, visualization and integrated discovery
IPA: Ingenuity Pathway Analysis
scRNA‐seq: single-cell RNA sequencing
DMEM: Dulbecco’s modified eagle medium
FBS: fetal bovine serum
SLC11A1: Solute Carrier Family 11 Member 1
ADRA2B: adrenoceptor alpha 2B: Adrenoceptor Alpha 2B
UCK2: uridine-cytidine kinase 2
MARCKSL1: MARCKS like 1
NEK2: NIMA related kinase 2
CHEK2: checkpoint kinase 2
DTYMK: deoxythymidylate kinase
PRC1: protein regulator of cytokinesis 1
EPCAM: epithelial cell adhesion molecule
POLR2L: RNA polymerase II, I and III subunit L
PPP2R1A: protein phosphatase 2 scaffold subunit alpha
POLR2G: RNA polymerase II subunit G
ITGB1BP1: integrin subunit beta 1 binding protein
ESR1: estrogen receptor 1
VEGA: vascular endothelial growth factor A
NMS: nodal metastasis status
TOP2A: DNA topoisomerase II alpha
UBE2C: ubiquitin conjugating enzyme E2 C
CENPN: centromere protein N
TUBB: tubulin beta class I
UBE2T: ubiquitin conjugating enzyme E2 T
PRC2: protein regulator of cytokinesis 2
ADRB2: adrenoceptor beta 2
DAB2IP: DAB2 interacting protein
CancerSCEM: Cancer Single-cell Expression Map.
