# Corrigendum: Single-cell RNA sequencing reveals the role of phosphorylation-related genes in hepatocellular carcinoma stem cells

**DOI:** 10.3389/fcell.2023.1045260

**Published:** 2023-10-23

**Authors:** Fuwen Yao, Yongqiang Zhan, Changzheng Li, Ying Lu, Jiao Chen, Jing Deng, Zijing Wu, Qi Li, Yi’an Song, Binhua Chen, Jinjun Chen, Kuifeng Tian, Zuhui Pu, Yong Ni, Lisha Mou

**Affiliations:** ^1^ Department of Hepatopancreatobiliary Surgery, Shenzhen Institute of Translational Medicine, Health Science Center, Shenzhen Second People’s Hospital, The First Affiliated Hospital of Shenzhen University, Shenzhen, China; ^2^ Shenzhen Xenotransplantation Medical Engineering Research and Development Center, Shenzhen Institute of Translational Medicine, The First Affiliated Hospital of Shenzhen University, Shenzhen Second People’s Hospital, Shenzhen, China; ^3^ Key Laboratory of Stem Cells and Tissue Engineering, Zhongshan School of Medicine, Sun Yat-sen University, Ministry of Education, Guangzhou, China; ^4^ Imaging Department, Shenzhen Institute of Translational Medicine, Health Science Center, Shenzhen Second People’s Hospital, The First Affiliated Hospital of Shenzhen University, Shenzhen, China

**Keywords:** AURKA, EZH2, tyrosine kinase inhibitors, TKI, protein kinases, cell cycle, single-cell RNA sequencing, hepatocellular carcinoma

In the published article, there was an error in Figure 7C as published. The red label and black label were reversed. The corrected Figure 7C and its caption is as follows: “**(C)** Hep3B and Huh7 cells were treated with gambogenic acid (EZH2 inhibitor, 2 μM) or alisertib (AURKA inhibitor, 10 μM) for 0, 12, 24, 32, 48, and 60 h, and cell viability was determined by Calcein-AM/PI staining assays.”

**FIGURE 7:**
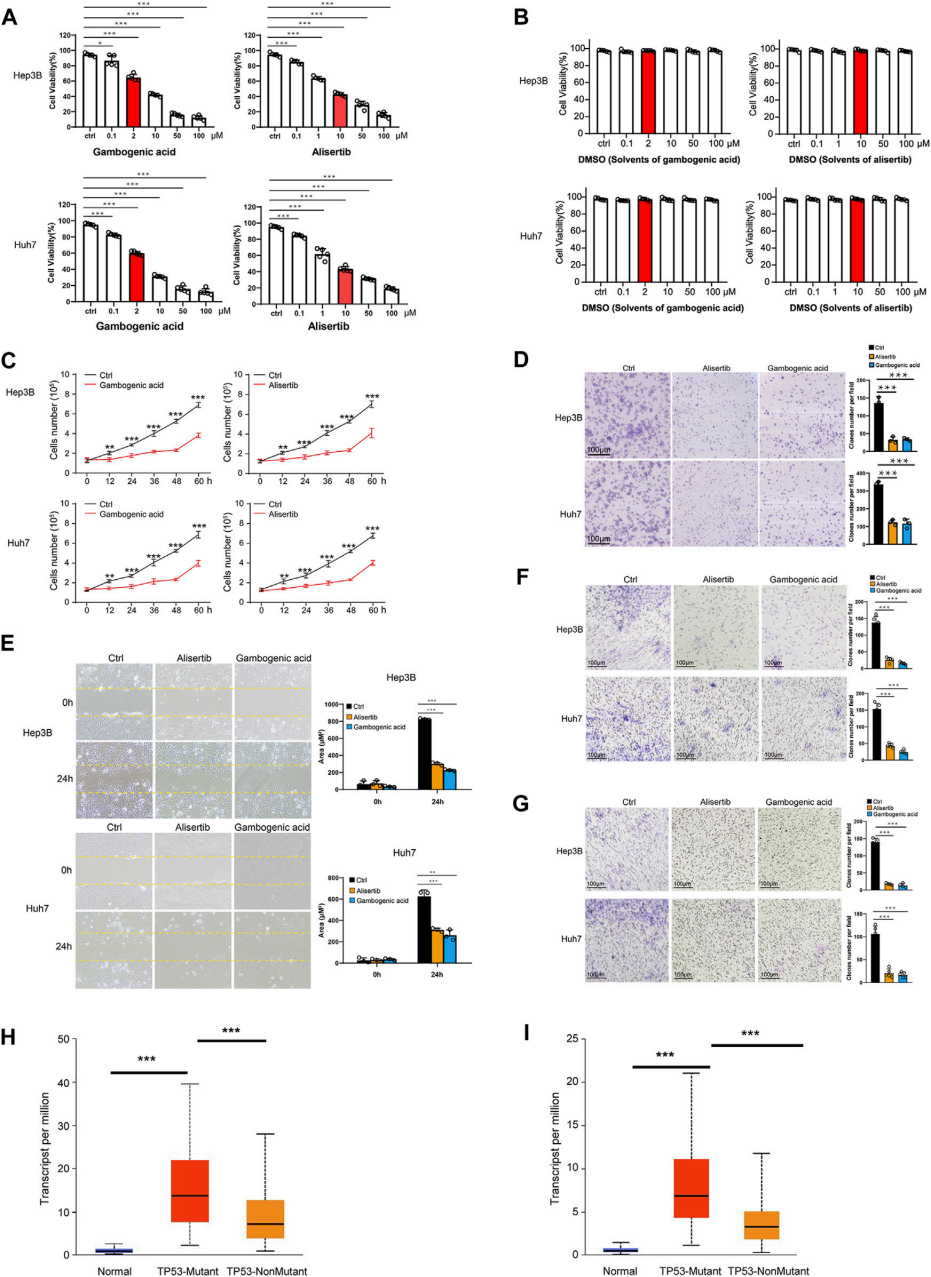
Gambogenic acid (EZH2 inhibitor) and alisertib (AURKA inhibitor) inhibit HCC cell proliferation, migration, and invasion. **(A)** Hep3B and Huh7 cells were treated with gambogenic acid (EZH2 inhibitor) or alisertib (AURKA inhibitor) at different concentrations (0–100 μM) for 48 h, and cell viability was determined by Calcein–AM/PI staining assays. **(B)** Hep3B and Huh7 cells were treated with DMSO (solvents of gambogenic acid and alisertib) at different concentrations (0–100 μM) for 48 h, and cell viability was determined by Calcein–AM/PI staining assays. **(C)** Hep3B and Huh7 cells were treated with gambogenic acid (EZH2 inhibitor, 2 μM) or alisertib (AURKA inhibitor, 10 μM) for 0, 12, 24, 32, 48, and 60 h, and cell viability was determined by Calcein–AM/PI staining assays. **(D)** Colony formation assays were conducted to analyze Hep3B and Huh7 cell proliferation with gambogenic acid (2 μM) or alisertib (10 μM) treatment. **(E, F)** Wound healing assays **(E)** and Transwell assays **(F)** were performed to detect the cell migratory abilities of Hep3B and Huh7 cells treated with gambogenic acid (2 μM) or alisertib (10 μM). **(G)** Transwell assays were performed to detect the cell invasion abilities of Hep3B and Huh7 cells treated with gambogenic acid (2 μM) or alisertib (10 μM). Data are expressed as the means ± s.d. Differences were considered statistically significant if p < 0.05. ns, no significance, *p < 0.05, **p < 0.01, ***p < 0.001. **(H, I)** Expression of AURKA **(H)** and EZH2 **(I)** in TCGA–LIHC based on TP3 mutation status.

The authors apologize for this error and state that this does not change the scientific conclusions of the article in any way. The original article has been updated.

