# Supplementary figures and images for: Single-Cell RNA Sequencing Reveals the Role of Phosphorylation-Related Genes in Hepatocellular Carcinoma Stem Cells
Source: Front Cell Dev Biol. 2022 Jan 4;9:734287. doi: 10.3389/fcell.2021.734287 (PMC8763978; doi:10.3389/fcell.2021.734287)

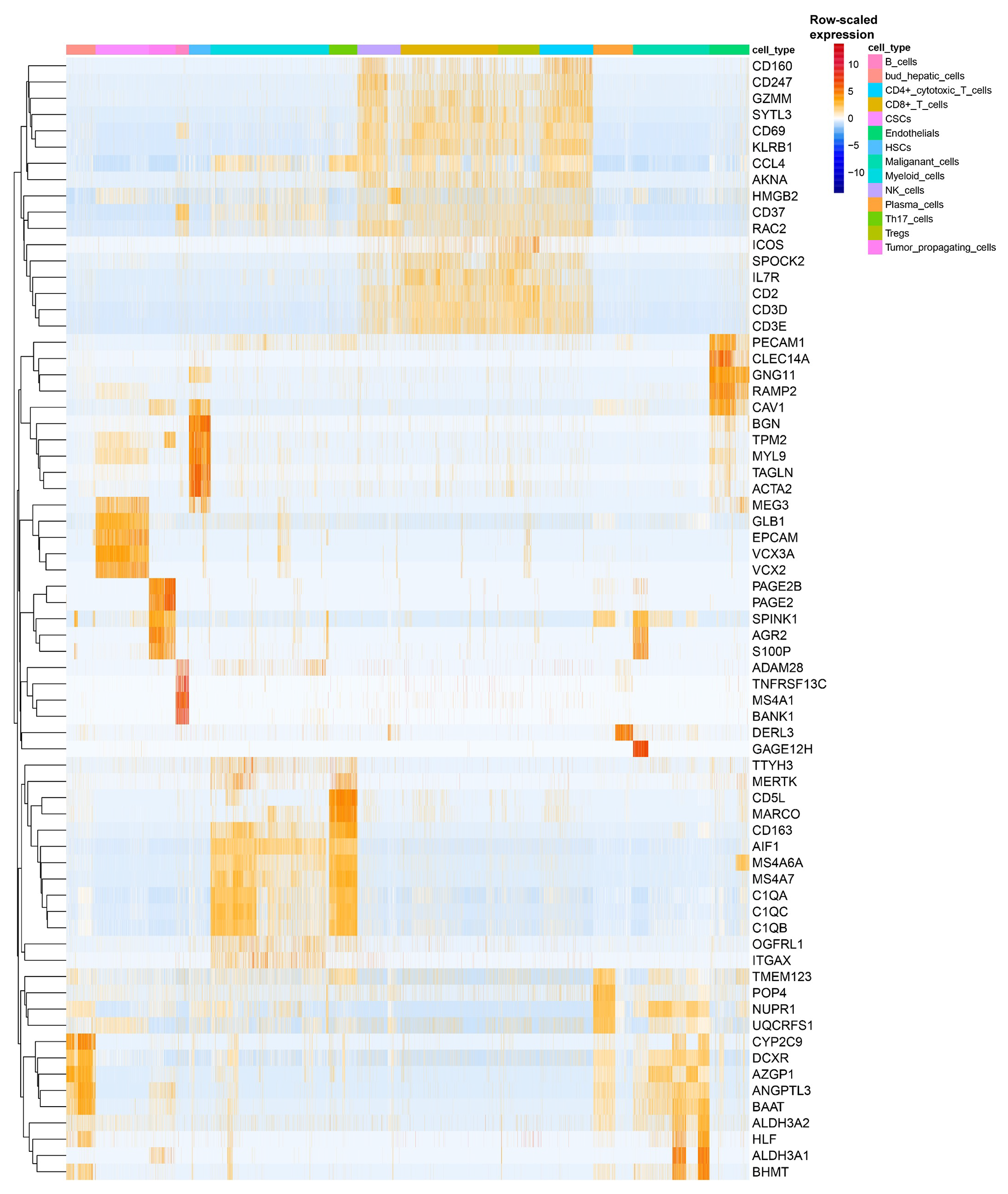

Supplement: Supplementary file 4 [file Image3.tif]

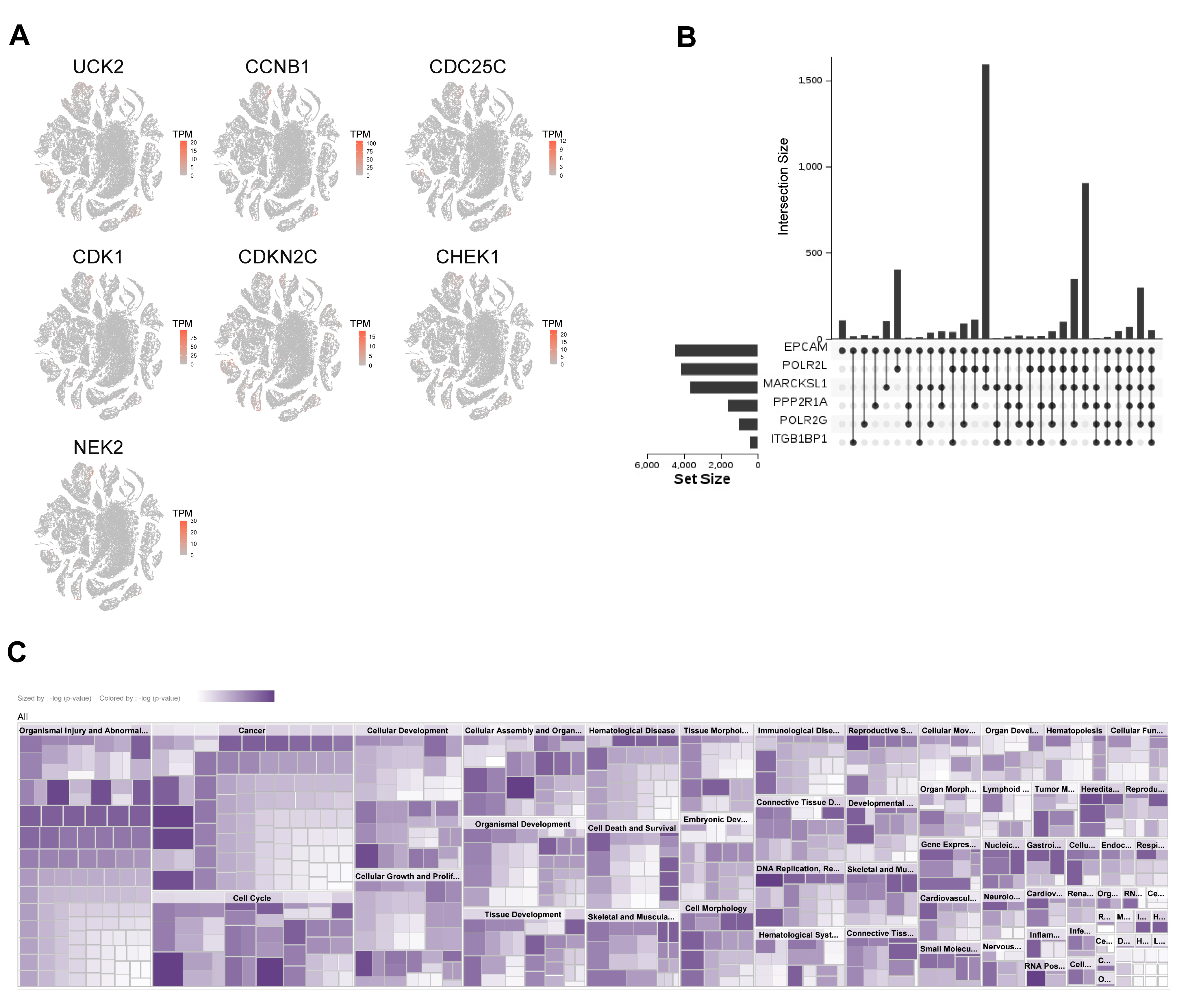

Supplement: Supplementary file 5 [file Image4.tif]

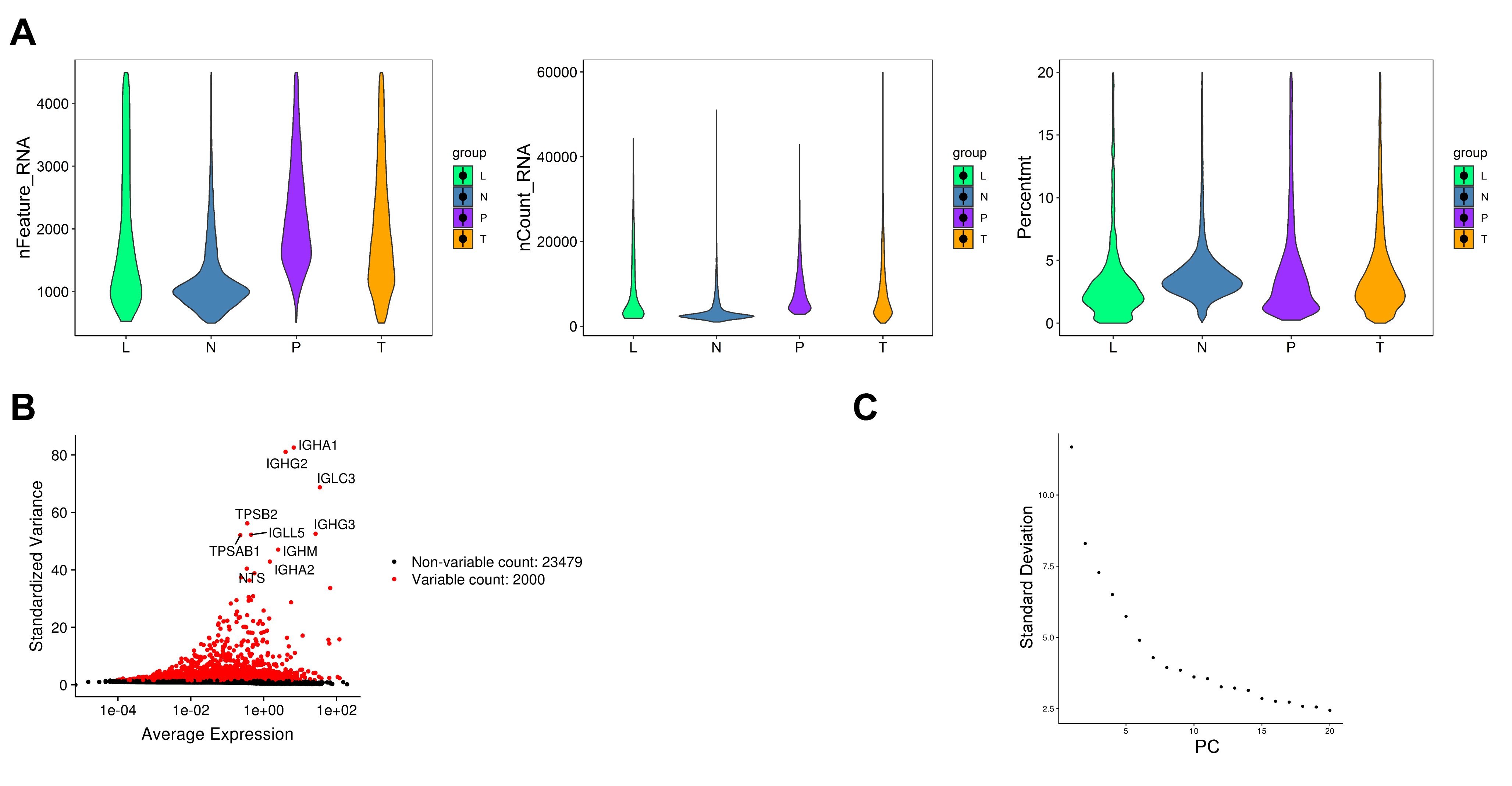

Supplement: Supplementary file 6 [file Image2.tif]

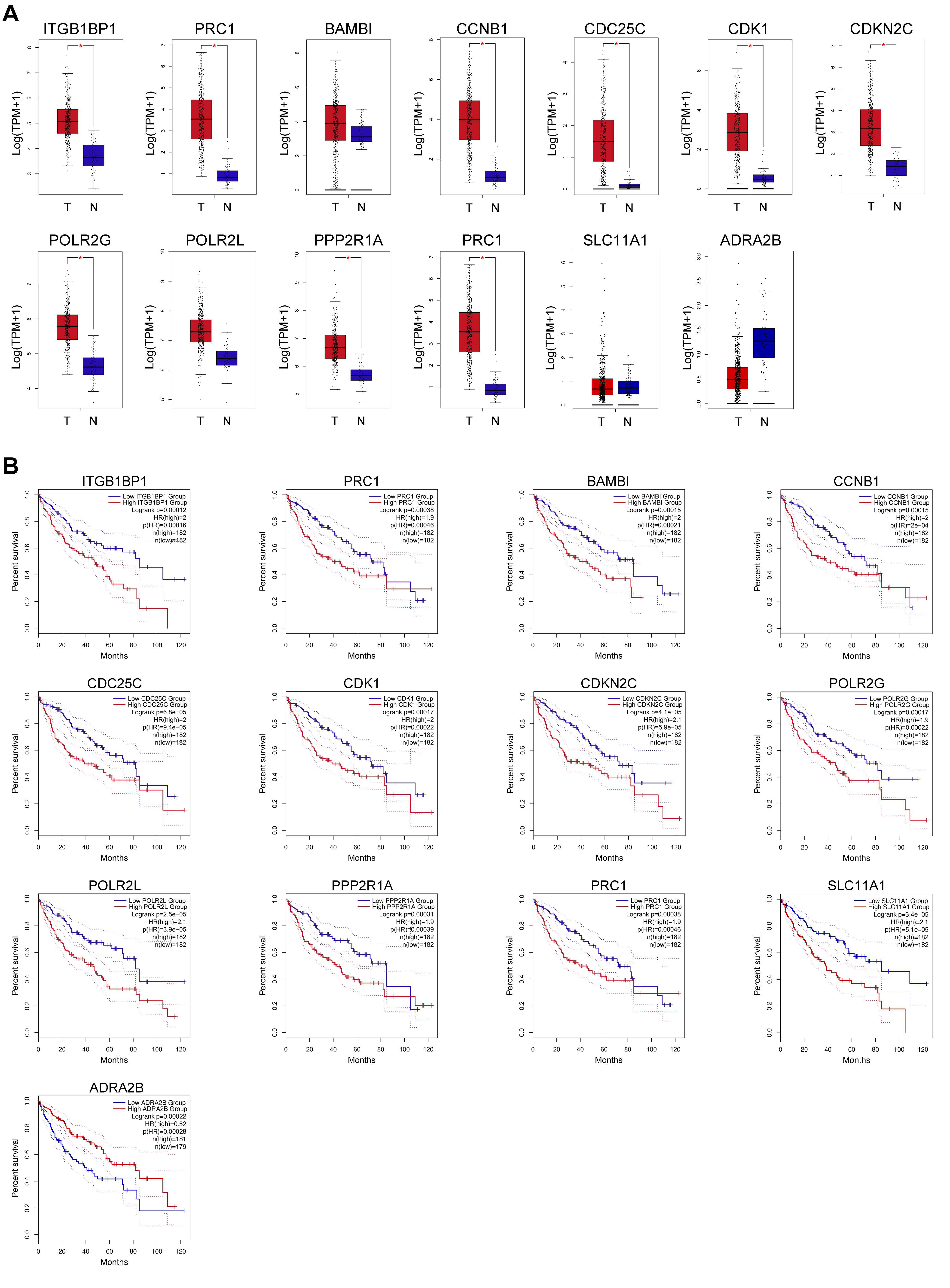

Supplement: Supplementary file 7 [file Image1.tif]

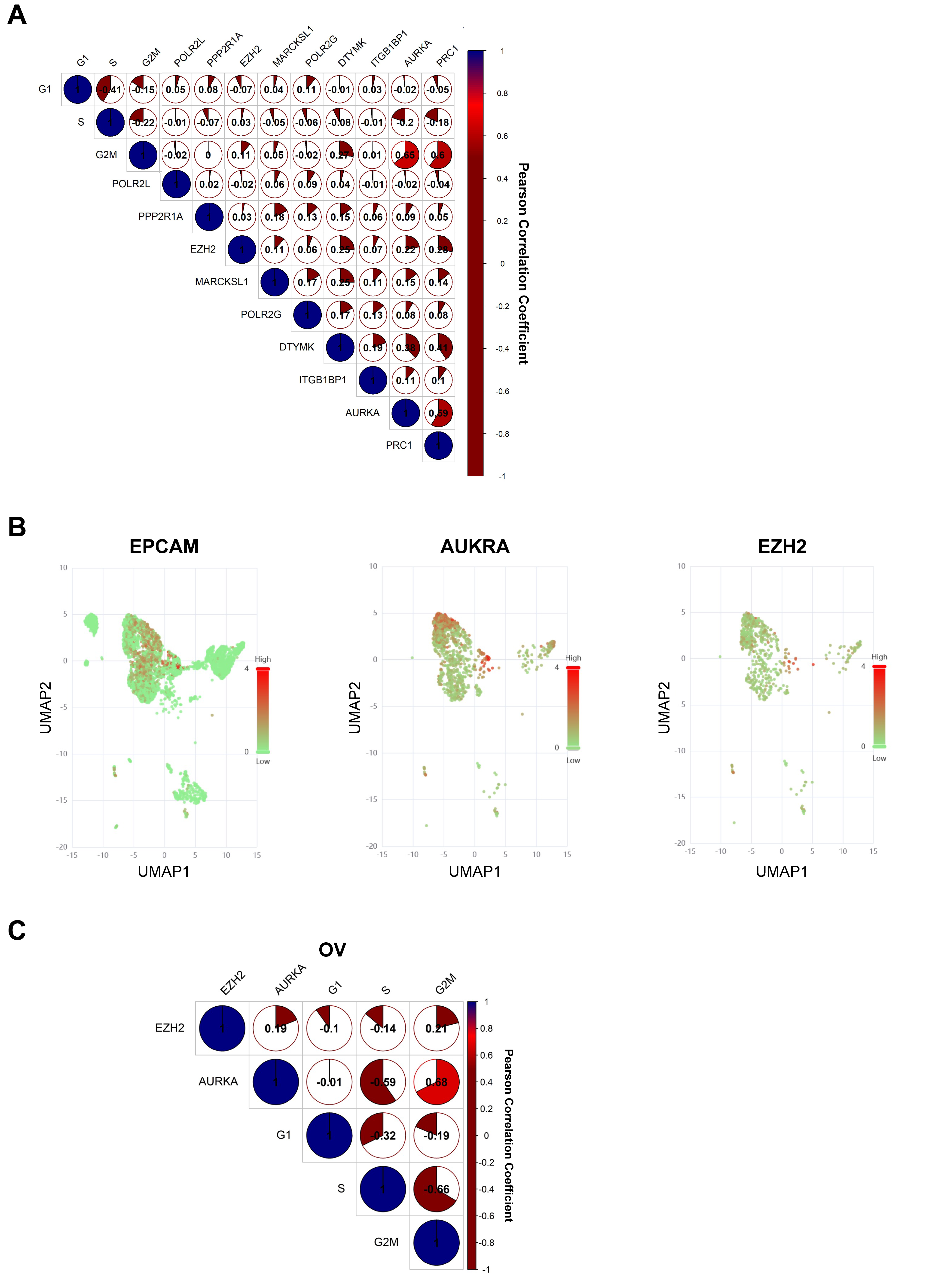

Supplement: Supplementary file 12 [file Image5.tif]
